# Benzodiazepine and zolpidem prescriptions during autologous stem cell transplantation

**DOI:** 10.1002/jha2.148

**Published:** 2021-02-06

**Authors:** Rahul Banerjee, Ann A. Lazar, Lisa Dunn, Jennifer Knoche, Mimi Lo, Shagun Arora, Sandy W. Wong, Jeffrey L. Wolf, Thomas G. Martin, Anand Dhruva, Nina Shah

**Affiliations:** ^1^ Division of Hematology/Oncology Department of Medicine University of California San Francisco San Francisco California; ^2^ Division of Oral Epidemiology Division of Biostatistics, and Biostatistics Core Helen Diller Family Comprehensive Cancer Center Department of Preventive and Restorative Dental Sciences and Department of Epidemiology and Biostatistics University of California San Francisco San Francisco California; ^3^ Division of Hematology/Oncology Department of Pharmacy University of California San Francisco San Francisco California

## Abstract

Multiple myeloma patients undergoing autologous stem cell transplantation (ASCT) may receive benzodiazepine or zolpidem‐class (B/Z) medications despite their risks in older patients. Of 205 myeloma patients (36% aged 65+) who underwent ASCT at our institution between 2017 and 2018, we found that B/Z prescription rates for anxiety/insomnia rose significantly from 26% before ASCT to 38% at discharge and 39% at Day +100. B/Z initiation while hospitalized was a strong predictor of B/Z persistence at Day +100. Our findings highlight the role of these potentially inappropriate medications during hospitalizations for ASCT, a period where nonpharmacologic strategies for managing anxiety/insomnia may be feasible.

In September 2020, the US Food and Drug Administration (FDA) issued a new boxed warning about long‐term risks of benzodiazepine medications such as physical dependence because of withdrawal symptoms. Similarly, Z‐class medications such as zolpidem carry an existing FDA boxed warning about the risk of sustaining serious injuries. Additional toxicities of benzodiazepines and zolpidem‐class (B/Z) medications in older adults include cognitive impairment, fractures, and motor vehicle accidents; as a result, both American and European guidelines recommend against their usage among adults aged ≥65 [1, 2]. We have anecdotally noted that patients with multiple myeloma (MM) undergoing autologous stem cell transplantation (ASCT) at our institution, many of whom are aged ≥65, are frequently prescribed B/Z medications for anxiety for insomnia during their inpatient hospitalizations for high‐dose melphalan chemotherapy followed by autologous stem cell rescue. On one level, this observation is understandable given the acute life interruptions and sudden physical limitations that ASCT entails. However, MM patients generally can reattain their baseline quality of life within 1‐2 months of ASCT and can expect to live several years after ASCT [3]. Although the long‐term risks of B/Z medications among older adults are well documented, usage patterns for B/Z medications among MM patients actively undergoing and recovering from ASCT are not well characterized.

We thus performed a single‐center retrospective cohort analysis of all adult MM patients who underwent ASCT at our institution during a 2‐year period between January 2017 and December 2018 (including outpatient ASCT recipients). We used clinician‐completed medication reconciliations to identify B/Z medications at three timepoints for each patient: (a) pre‐ASCT hospital admission note, or most recent premelphalan note for outpatient recipients; (b) hospital discharge summary, or Day (D) +14 clinic note for outpatient recipients; and (c) D +100 clinic note. Lorazepam prescriptions specifically for as‐needed management of nausea/vomiting were excluded. We used two‐sided McNemar's tests (with alpha .05) to compare proportions of patients with ≥1 B/Z prescription at each timepoint. We used longitudinal models to assess whether B/Z prescription rates differed across time according to pre‐ASCT lines of therapy (≥2 vs 1), age at ASCT (≥65 vs <65), melphalan conditioning (200 vs 140 mg/m^2^), and ASCT setting (inpatient vs outpatient); proportions between subgroups were compared using an interaction term from the logistic regression model with the presence versus absence of ≥1 B/Z prescription at D +100 as the dichotomous primary endpoint. Lastly, for the subset of patients who were B/Z‐naïve before ASCT, we compared patient characteristics based on the presence versus absence of ≥1 B/Z prescription at D +100 using Fisher's exact test with *P* < .05 defining significance. For these B/Z‐naïve patients, we calculated odds ratios for the presence of ≥1 B/Z prescription at D +100 based on the presence or absence of ≥1 B/Z prescription at hospital discharge. Data were analyzed using Stata (StataCorp, College Station, TX) or SAS v.9.4 (SAS, Cary, NC).

We identified a total of 205 patients (43% female) who underwent ASCT at our institution; no tandem ASCTs were performed. The median age at ASCT was 61, and 73 patients (36%) were aged 65 or older. For patients who underwent inpatient ASCT (90%, n = 184), the median date of discharge was D +14 after ASCT. Fifty‐eight percent (n = 118) of patients received no B/Z medications for anxiety/insomnia during the study period, 30% (n = 62) received a single B/Z medication at ≥1 timepoint, while 12% (n = 25) were prescribed 2+ distinct B/Z medications over the 3‐month period. The most commonly used B/Z medications were lorazepam and zolpidem in 36% (n = 74) and 12% (n = 24) of patients, respectively. The proportion of patients with ≥1 B/Z prescription rose from 26% (n = 53) before ASCT to 38% (n = 78) at discharge, then 39% (n = 80) at D +100. The discharge and D +100 proportions of B/Z prescriptions were both significantly higher than the baseline pre‐ASCT proportion (*P* < .001 in both cases).

As shown in Figure 1, individual B/Z trajectories over time fell into one of six patterns. Longitudinal modeling confirmed statistically significant increases in the proportion of patients with ≥1 B/Z prescription based on timepoint (*F*‐statistic 12.31, *P* < .01) but not based upon age, prior lines of therapy, melphalan dosing, or inpatient versus outpatient ASCT. We then specifically analyzed the 152 patients (74% of the entire cohort) who were B/Z‐naïve before ASCT. Twenty percent of these patients (n = 30) had ≥1 B/Z prescription present at D +100: 27 patients were prescribed lorazepam, eight patients were prescribed zolpidem, and five patients were prescribed both medications. As shown in Table 1, the cohorts of B/Z‐naïve patients (ie, ≥1 B/Z prescription present at D +100 vs absent at D +100) were similar in terms of demographic and clinical characteristics. However, 84% of B/Z‐naïve patients who had ≥1 B/Z prescription at hospital discharge, then had ≥1 B/Z prescription at D +100; in contrast, for B/Z‐naïve patients who remained B/Z‐naïve at discharge, only 7% had ≥1 B/Z prescription present at D +100. The corresponding odds ratio for de novo B/Z initiation by hospital discharge predicting B/Z presence at D +100 was 11.9 (95% confidence interval 6.2‐22.8, *P *< .001).

**FIGURE 1:**
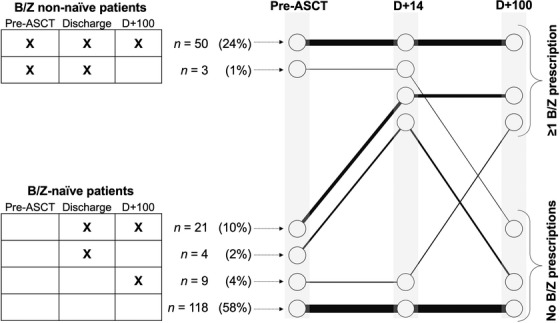
Longitudinal characterizations of B/Z prescriptions. Longitudinal trajectories of having ≥1 B/Z prescription at each timepoint, presented in tabular format on the left and in graphical format on the right. Abbreviations: ASCT, autologous stem cell transplantation; B/Z, benzodiazepine and zolpidem‐class medications; D, day

**TABLE 1:** Characteristics of B/Z‐naïve patients

	Absent		Present		
B/Z prescriptions at D +100	n	%	n	%	*P*‐value
Overall	122	80	30	20	
Age at ASCT					1.0
<65	81	80	20	20	
≥65	41	80	10	20	
Gender					.15
Male	77	85	14	15	
Female	45	74	16	26	
Prior therapies					.66
<2 Lines	87	81	20	19	
2+ Lines	35	78	10	22	
Melphalan dose					1.0
200 mg/m^2^	106	80	26	20	
140 mg/m^2^	16	80	4	20	
ASCT setting					.33
Inpatient	110	81	25	19	
Outpatient	12	71	5	29	
B/Z prescriptions at discharge					**<.01** *
Absent	118	93	9	7	
Present	4	16	21	84	

*Note*. Only patients who were B/Z‐naïve (defined as the absence of any B/Z prescriptions before ASCT) are shown.

Abbreviations: ASCT, autologous stem cell transplantation; B/Z, benzodiazepine or zolpidem‐class medications; D, day; mg/m^2^, milligrams per square meter.

* Significant with *P* < .05.

To our knowledge, our study represents the first attempt at quantitating B/Z medication usage among predominantly older patients with MM during the intensive peri‐ASCT period. Even after excluding lorazepam prescriptions for nausea/vomiting, we nevertheless found that B/Z prescription rates among MM patients rose rapidly and significantly from 26% to 38% within 2 weeks of ASCT. Consistent with the FDA's recently issued boxed warning about the persistence of B/Z medications once initiated, we found that the presence of a de novo B/Z prescription at hospital discharge among previously B/Z‐naïve patients conferred an almost 12‐fold risk of its continued presence at D +100. In contrast, the symptoms of anxiety or insomnia that would typically prompt B/Z initiation generally resolve within weeks of ASCT [4]. While we did not directly characterize the toxicities of B/Z medications in our retrospective chart‐based study, these risks in MM patients include ambulatory falls and fractures [5]. Long‐term B/Z medications may contribute to polypharmacy as well, which is a major problem for MM patients and may contribute to nonadherence with important post‐ASCT medications such as lenalidomide [5, 6, 7].

Limitations of our retrospective single‐center analysis include our reliance on the dichotomous presence or absence of B/Z prescriptions as recorded within clinician‐completed medication reconciliations at each visit. As such, we are unable to ascertain how frequently outpatient B/Z prescriptions were actually taken by patients. Given our chart‐based methodology and the small absolute number of B/Z‐naïve patients who were started on B/Z prescriptions at D +100, we are unable to assess for confounders including symptom burden that might have explained an association between B/Z initiation at discharge and B/Z presence at D +100. Future steps for our group include prospective validation of our findings through patient‐reported outcome assessments of anxiety and insomnia alongside questionnaires about medication usage and patient falls. Regardless, our data suggest that the ideal timeframe for targeting B/Z medication prescription usage is during the index hospitalization for ASCT (which corresponds to the peak of patient symptoms and also the nadir of patient coping mechanisms). As such, we are now also working with nursing staff and inpatient providers at our institution to identify workflows that may lead to the initiation of B/Z medications for sleep or anxiety during ASCT hospitalizations.

Given that our field is increasingly recognizing the risks of B/Z medications in older adults, other strategies to manage anxiety and insomnia in a safer manner during stem cell transplantation are warranted. In its Drug Safety Communication about benzodiazepines, the FDA highlighted the role of complementary and integrative treatments in the management of anxiety and insomnia. Several integrative strategies have been shown to be feasible during ASCT hospitalizations, for example: (a) music therapy, with patient‐selected live music performances and patient participation, if possible; (b) acupuncture needle insertion into the skin by trained acupuncturists; and even (c) programmed environmental illumination, with circadian patterns of bright white lighting within patient rooms [8, 9, 10, 11, 12, 13]. In randomized studies, music therapy and acupuncture have additionally been shown to have “pharmacomimetic” effects with regard to lowering the amount of narcotic medications that patients require while they are hospitalized for ASCT [11, 12]. While these types of integrative interventions are not yet recommended by society guidelines (nor are they routinely available at most institutions, including ours), we believe that nonpharmacologic interventions have minimal risks during ASCT—including acupuncture, which is safe for thrombocytopenic cancer patients [14]—and thus warrant further study. Indeed, by offsetting B/Z medication initiation and possibly reducing long‐term psychiatric complications after ASCT (which include posttraumatic stress disorder) [15], similar intrahospitalization interventions for anxiety and insomnia may offer durable benefits to MM patients well beyond ASCT.

## FUNDING INFORMATION

Publication made possible in part by support from the UCSF Open Access Publishing Fund.

[Correction added on 24 February 2021, after first online publication funding information has been added].

## Data Availability

The data that support the findings of this study are available on request from the corresponding author. The data are not publicly available due to privacy or ethical restrictions.
